# Pediatric hospital utilization for patients with avoidant restrictive food intake disorder

**DOI:** 10.1186/s40337-024-00996-z

**Published:** 2024-03-25

**Authors:** Carly E. Milliren, McGreggor Crowley, Julia K. Carmody, Elana M. Bern, Olivia Eldredge, Tracy K. Richmond

**Affiliations:** 1 Institutional Centers for Clinical and Translational Research, Boston Children’s Hospital, 300 Longwood Avenue, Boston, MA 02115 USA; 2 Division of Gastroenterology, Hepatology, and Nutrition, Boston Children’s Hospital, Boston, MA USA; 3 Department of Pediatrics, Harvard Medical School, Boston, MA USA; 4 Department of Psychiatry, Harvard Medical School, Boston, MA USA; 5 Division of Adolescent and Young Adult Medicine, Boston Children’s Hospital, Boston, MA USA

**Keywords:** Avoidant restrictive food intake disorder (ARFID), Pediatric hospitals, Inpatient hospitalization, Hospital utilization, 30-day readmissions

## Abstract

**Background:**

Avoidant restrictive food intake disorder (ARFID) is a relatively new feeding and eating disorder added to the DSM-5 in 2013 and ICD-10 in 2018. Few studies have examined hospital utilization for patients with ARFID specifically, and none to date have used large administrative cohorts. We examined inpatient admission volume over time and hospital utilization and 30-day readmissions for patients with ARFID at pediatric hospitals in the United States.

**Methods:**

Using data from the Pediatric Health Information System (PHIS), we identified inpatient admissions for patients with ARFID (by principal *International Classification of Diseases, 10th Revision,* ICD-10 diagnosis code) discharged October 2017–June 2022. We examined the change over time in ARFID volume and associations between patient-level factors (e.g., sociodemographic characteristics, co-morbid conditions including anxiety and depressive disorders and malnutrition), hospital ARFID volume, and hospital utilization including length of stay (LOS), costs, use of enteral tube feeding or GI imaging during admission, and 30-day readmissions. Adjusted regression models were used to examine associations between sociodemographic and clinical factors on LOS, costs, and 30-day readmissions.

**Results:**

Inpatient ARFID volume across n = 44 pediatric hospitals has increased over time (β = 0.36 per month; 95% CI 0.26–0.46; *p* < 0.001). Among N = 1288 inpatient admissions for patients with ARFID, median LOS was 7 days (IQR = 8) with median costs of $16,583 (IQR = $18,115). LOS and costs were highest in hospitals with higher volumes of ARFID patients. Younger age, co-morbid conditions, enteral feeding, and GI imaging were also associated with LOS. 8.5% of patients were readmitted within 30 days. In adjusted models, there were differences in the likelihood of readmission by age, insurance, malnutrition diagnosis at index visit, and GI imaging procedures during index visit.

**Conclusions:**

Our results indicate that the volume of inpatient admissions for patients with ARFID has increased at pediatric hospitals in the U.S. since ARFID was added to ICD-10. Inpatient stays for ARFID are long and costly and associated with readmissions. It is important to identify effective and efficient treatment strategies for ARFID in the future.

**Supplementary Information:**

The online version contains supplementary material available at 10.1186/s40337-024-00996-z.

## Abstract (plain language summary)

Recent studies indicate that Avoidant Restrictive Food Intake Disorder (ARFID) is a complex feeding and eating disorder often diagnosed in younger children. To date, there are no large studies using administrative data to examine hospital utilization or costs among patients with ARFID. In a geographically diverse cohort of pediatric hospitals in the United States, we found inpatient admissions for ARFID have increased over time and that ARFID is associated with long, costly stays and readmissions which has important implications for identifying efficient treatment strategies. Future studies are needed to explore effective and efficient treatment strategies and prevent readmissions in this patient population.

**Supplementary Information:**

The online version contains supplementary material available at 10.1186/s40337-024-00996-z.

## Introduction

Avoidant Restrictive Food Intake Disorder (ARFID) is a relatively new feeding and eating disorder characterized by food refusal or restriction of intake that is thought to be unrelated to weight or body image disturbance [1, 2]. Patients with ARFID tend to be younger than those with other eating disorders and avoidance of foods may be driven by a number of factors including low appetite or lack of interest in food or eating, sensory sensitivities related to the appearance, color, or texture of the food, and/or a fear of aversive consequences such as vomiting or choking following food intake [1–3]. The ARFID diagnosis was added to the 5th edition of the *Diagnostic and Statistical Manual of Mental Disorders* (DSM-5) published in 2013 [4], and the *International Classification of Diseases, 10th Revision* (ICD-10) in 2018 (effective date October 1, 2017) [5]. Prior to inclusion in ICD-10, ARFID symptoms were captured in broader and non-specific categories of feeding disorders or behaviors making it difficult to examine ARFID-specific patterns over time using administrative data [6]. Despite inclusion in the DSM-5 and ICD-10, large-scale studies examining changes in prevalence or patterns of diagnosis are lacking. Existing studies have largely documented prevalence within specialized outpatient programs such as Gastroenterology or Eating Disorder programs or in particular populations, such as those with autism spectrum disorder [7–12]. It is important to understand changes in the use of the ARFID diagnosis and whether such changes are related to usage of a newly available diagnostic code, increased recognition, increased prevalence, or some combination of these factors. It is currently unknown whether the volume of inpatient ARFID discharges has increased over time with increasing awareness of the diagnosis, particularly in the context of increasing incidence of eating disorders more generally following the onset of the COVID-19 pandemic [13–17].

Patients with ARFID requiring hospitalization often have a complex presentation, frequently including malnutrition, significant mental health co-morbidities, and/or psychosocial factors such as adverse parental feeding styles and parental intrusiveness or other impacts to psychosocial functioning [1, 12, 18–20]. Common co-morbidities include mental health diagnoses such as generalized anxiety disorder or obsessive–compulsive disorder and neurodevelopmental diagnoses including autism spectrum disorder or attention-deficit hyperactivity disorder (ADHD) [1, 12, 21, 22]. Though less common, gastroenterological (GI) co-morbidities such as eosinophilic esophagitis or celiac disease may be present though the diagnosis of ARFID requires that feeding problems are out of proportion to what is expected in these disorders [12]. Because patients with ARFID often present with significant symptomatology, and because mental health co-morbidities can make it difficult to differentiate between physiologic or functional symptoms, patients often receive extensive and costly medical workups [12]. Due to the relatively recent development of ARFID diagnostic criteria and considerable variability in presentation, a wide range of management approaches have been developed for patients and/or caregivers, including cognitive-behavioral therapy for ARFID (CBT-AR) and interoceptive exposure treatment using the feeling and body investigators (FBI) framework, among others [23–28]. However, these approaches have been tested primarily in outpatient settings and findings related to efficacy or generalizability are limited given small studies to date, especially regarding protocolized treatment approaches in acute care settings for patients hospitalized with ARFID [12, 18, 23].

Though there is high risk for patients with ARFID to have prolonged hospitalizations with intensive and costly workup, data describing trends in volume, cost or length of stay (LOS) during medical hospitalizations for ARFID are limited, particularly in large cohorts. Given this paucity of literature, we set out to describe the monthly trend in usage of the ICD-10 ARFID diagnosis for inpatient hospital discharges as well as hospital utilization and factors associated with utilization among pediatric hospitalizations for ARFID using a large administrative dataset from a diverse group of tertiary care pediatric hospitals. Our first aim was to examine inpatient volume for patients with a principal diagnosis of ARFID in the nearly five years since ARFID was added as an ICD-10 diagnostic billing code. Our second aim was to examine sociodemographic characteristics, clinical factors, and hospital utilization (LOS, costs, 30-day readmissions) during pediatric ARFID hospitalizations overall and by hospital ARFID volume. Finally, for our third aim, we describe associations between sociodemographic and clinical factors with hospital utilization (LOS, costs, and 30-day readmissions) during pediatric ARFID hospitalizations. We had the following hypotheses: the volume of pediatric ARFID hospitalizations increased since inclusion in ICD-10 (Aim 1), hospitals treating more inpatients with ARFID have shorter stays, lower costs, and fewer readmissions (Aim 2), and co-morbid conditions including anxiety, depression, and other GI illnesses are associated with longer hospital stays (Aim 3).

## Methods

### Procedure

This study used administrative data from the Pediatric Health Information System (PHIS) which is a comparative database on inpatient discharges from 50 tertiary care pediatric hospitals across the United States compiled by the Children’s Hospital Association [29]. We included inpatient medical admissions for patients 3 years or older diagnosed with ARFID and discharged from PHIS hospitals from October 1, 2017 to June 30, 2022 and any inpatient readmissions through July 31, 2022. ARFID was identified by principal discharge diagnosis code (ICD-10 code F50.82—Avoidant/restrictive food intake disorder). We restricted our sample to patients 3 years of age or older at admission as ARFID is not uniformly diagnosed below preschool age in our experience and there were fewer than 20 ARFID discharges in PHIS for patients younger than 3 years. We restricted our analysis to medical admissions given clinical differences in patient populations between those admitted for medical versus psychiatric treatment which would likely impact hospital utilization. We excluded n = 298 discharges with any psychiatric treatment unit charges based on flags available in PHIS.

To examine the change in volume of ARFID discharges over time in our first aim, we included n = 44/50 hospitals submitting data for the full 57-month study time period (n = 70 discharges from 6 hospitals with incomplete data excluded). For comparison, we extracted aggregate volume of a) total inpatient discharges for all diagnoses, and b) all inpatient eating disorder discharges (all ICD-10 codes beginning with F50) at the included hospitals during the same period. The included hospitals were distributed across Census regions and divisions in the U.S.

In our analysis related to Aims 2 and 3 examining individual patient sociodemographic characteristics, clinical factors, and hospital utilization for ARFID, we excluded hospitals with fewer than 20 ARFID discharges over the study period (n = 182 discharges from 22 hospitals excluded) due to the potential for limited generalizability among hospitals with low ARFID volume. This cutoff was chosen as it corresponds to approximately four inpatient ARFID admissions annually over the nearly five-year study period. The remaining n = 22 hospitals included were distributed across regions and divisions of the U.S.

### Measures

We extracted sociodemographic information, hospital utilization, ICD-10 diagnosis and procedure codes, and Clinical Transaction Classification (CTC) codes for inpatient ARFID discharges and any associated readmissions within 30 days.

#### Sociodemographic characteristics

Sociodemographic characteristics included age, race and ethnicity, insurance payor, and median household income by ZIP code which was divided into three categories based on 2016 tertiles of income in the United States [30].

#### Co-morbid diagnoses

We identified co-morbid diagnoses of interest by examining frequencies of secondary ICD-10 diagnosis codes within our cohort as well as relying on our clinical experience with our hospital’s ARFID population. Co-morbid diagnoses included malnutrition, GI illnesses (e.g., gastro-esophageal reflux disease, inflammatory bowel disease), psychiatric diagnoses including mental health disorders (e.g., depressive disorder, anxiety disorders), and neurodevelopmental disorders (attention-deficit hyperactivity disorder [ADHD], autism spectrum disorder). The full listing of diagnosis codes used to identify these co-morbid conditions and diagnoses can be found in Additional File 1: Supplementary Appendix A.

#### Hospital utilization

Measures of hospital utilization extracted included LOS, costs, and intensive care unit (ICU) utilization. Standardized costs for each discharge are provided by PHIS and derived from charges converted to (direct and indirect) costs according to hospital-specific ratios of costs-to-charges, adjusted for geographic region using Centers for Medicare and Medicaid Services wage and price index, and standardized to eliminate between and within-hospital cost variation for individual items or services [31]. Standardization is based on a cost master index derived from median-of-hospital-median-costs for every CTC coded item or service [31]. Total standardized cost for each discharge is calculated as the sum of cost master index-based cost times the number of units for each CTC code for all items or services provided with a valid CTC code and cost available in the cost master index [31].

#### Enteral tube feeding

Using ICD-10 procedure codes and CTC codes for clinical services, supply, and/or other transacted services, we identified patients who received enteral tube feeding at any point during admission (see Additional File 1: Supplementary Appendix B). The included codes were those for feeding tube placement or use and were inclusive of nasogastric, nasojejunal, or nasoduodenal tube feeding as well as surgically-placed methods including gastrostomy or jejunostomy.

#### GI imaging and surgical procedures

We also identified patients undergoing GI imaging procedures including colonoscopy, endoscopy, and other GI or nutrition imaging (e.g., GI fluoroscopy, abdominal CT scan or MRI, GI tract ultrasound, bone age x-ray) by ICD-10 procedure codes and imaging CTC codes (see Additional File 1: Supplementary Appendix C). Patients with any surgical procedure during admission were identified by PHIS flags capturing any billed charges for operating room services using CTC codes.

#### Hospital ARFID volume

We constructed a 3-category variable for hospital-level ARFID volume based on quartiles of discharge volume for patients with a principal ARFID diagnosis over the study period, dividing hospitals into: low volume (bottom quartile), medium volume (middle 50%), and high volume (top quartile).

#### Reason for readmission

We used principal ICD-10 diagnosis and procedure codes to classify primary reason for readmission.

### Statistical analysis

To examine the change over time in ARFID discharge volume in our first aim, we used unadjusted time series regression to examine the aggregate total monthly trend in volume across hospitals. We report the slope (β) for the monthly trend along with the standard error (SE), 95% confidence interval (CI), and *p* value. To account for any underlying changes in overall hospital volume during the same period, we also examined the monthly trend in ARFID discharges indexed to a) total inpatient discharges and b) total eating disorder inpatient discharges, calculating the volume of ARFID discharges per 10,000 inpatient discharges and per 100 eating disorder discharges.

In our patient-level analyses for Aims 2 and 3, we report mean (standard deviation; SD) or median (interquartile range; IQR) for continuous variables and frequency (percent) for categorical variables. For Aim 2, we examined distributions of sociodemographic characteristics, clinical factors, and hospital utilization by hospital volume category using one-way ANOVA or Kruskal–Wallis tests for continuous variables and chi-square tests for categorical variables. For Aim 3, we used adjusted regression models to examine associations between sociodemographic characteristics and clinical factors with LOS using Poisson regression, costs using gamma regression, and 30-day readmissions using logistic regression. We report adjusted risk ratios (aRR) and 95% confidence intervals (CI) for LOS and costs and adjusted odds ratios (aOR) and 95% CI for readmissions.

All analyses were performed using SAS (version 9.4; Cary, NC) and *p* < 0.05 was considered statistically significant.

## Results

### Inpatient ARFID volume over time (Aim 1)

**Fig. 1:**
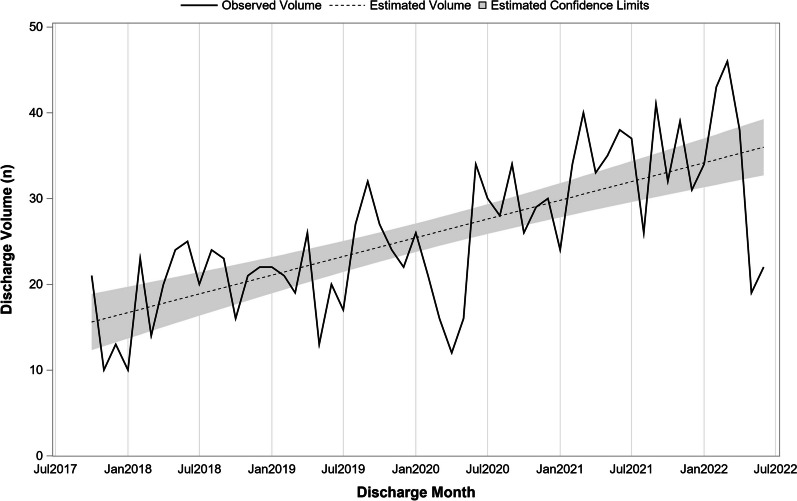
Monthly discharge volume for patients with ARFID from October 2017 to June 2022. Data from 44 Pediatric Health Information System hospitals (N = 1470 discharges over 57 months). Results from unadjusted time series regression analysis examining the monthly trend in ARFID discharge volume

From October 2017 to the end of our study period in June 2022, there were a total of 1470 inpatient discharges with a principal diagnosis of ARFID across 44 hospitals. ARFID discharges increased significantly over time (β = 0.36 per month; SE = 0.05; 95% CI 0.26–0.46; *p* < 0.001; Fig. 1). Monthly discharge volume approximately doubled from an estimated 15.6 in October 2017 to 36.0 in June 2022. The monthly trend was similar (β = 0.36 per month; SE = 0.12; 95% CI 0.10–0.62; *p* = 0.008) prior to March 2020 and the onset of the COVID-19 pandemic. Indexing to total inpatient discharges also indicated ARFID volume increased over time (β = 0.10 per 10,000 discharges per month; SE = 0.02; 95% CI 0.07–0.13; *p* < 0.001) even when accounting for overall patient volume. Relative to all eating disorder discharges, ARFID volume has remained stable over time (β = 0.03 per 100 ED discharges per month; SE = 0.02; 95% CI − 0.027 to 0.08; *p* = 0.310).

### Sociodemographic and clinical factors and hospital utilization (Aim 2)

After additional exclusions for very low hospital volume, we included 1288 ARFID discharges from 22 hospitals in our analysis examining patient-level factors and hospital utilization. Per hospital, the median number of discharges was 46.5 (range 22–140). By hospital ARFID volume, the median number of discharges was 26 per hospital (range 22–31) among n = 5 low volume hospitals, 49 (range 32–69) among n = 13 medium volume, and 127 (range 86–140) among n = 4 high volume. Table 1 shows demographic and clinical characteristics of patient discharges by hospital ARFID volume. Average age was 13.9 years (SD = 3.9) with the majority among patients who were female sex, white non-Hispanic race, and with private insurance. There were significant differences by hospital volume in the distribution of sociodemographic and clinical factors. 1044 (81.1%) patients had at least one co-morbid medical diagnosis and such diagnoses were more likely for discharges from higher volume hospitals (*p* = 0.019). Malnutrition was the most common medical comorbidity (n = 969; 75.2%). Co-morbid diagnoses for other GI conditions were rare with the exception of gastro-esophageal reflux disease. Psychiatric diagnoses were very common with 1053 (81.8%) patients having at least one co-morbid psychiatric diagnosis.

**Table 1:** Sociodemographic characteristics for patients hospitalized with ARFID at 22 PHIS hospitals (N = 1288)

	n (%)				*p* value
	Overall (N = 1288; 22 hospitals)	Hospital ARFID Volume			
		Low (n = 132; 5 hospitals)	Medium (n = 676; 13 hospitals)	High (n = 480; 4 hospitals)	
*Sociodemographic factors*					
Age (years), *mean (SD)*	13.9 (3.9)	13.1 (3.7)	13.7 (4.0)	14.4 (3.6)	< 0.001
Age category					< 0.001
3–7 years	112 (8.7%)	11 (8.3%)	73 (10.8%)	28 (5.8%)	
8–11 years	259 (20.1%)	40 (30.3%)	123 (18.2%)	96 (20.0%)	
12–17 years	781 (60.6%)	78 (59.1%)	417 (61.7%)	286 (59.6%)	
18 + years	136 (10.6%)	3 (2.3%)	63 (9.3%)	70 (14.6%)	
Female sex	903 (70.1%)	100 (75.8%)	474 (70.1%)	329 (68.5%)	0.276
Race and ethnicity					< 0.001
White, non-Hispanic	886 (68.8%)	80 (60.6%)	486 (71.9%)	320 (66.7%)	
Hispanic	159 (12.3%)	28 (21.2%)	81 (12.0%)	50 (10.4%)	
Black/African-American, non-Hispanic	77 (6.0%)	6 (4.6%)	38 (5.6%)	33 (6.9%)	
Asian or Pacific Islander	41 (3.2%)	3 (2.3%)	25 (3.7%)	13 (2.7%)	
Another race, non-Hispanic	54 (4.2%)	3 (2.3%)	21 (3.1%)	30 (6.3%)	
Multiple race, non-Hispanic	24 (1.9%)	3 (2.3%)	7 (1.0%)	14 (2.9%)	
Unknown	47 (3.7%)	9 (6.8%)	18 (2.7%)	20 (4.2%)	
Insurance payor					< 0.001
Private	762 (59.2%)	65 (49.2%)	396 (58.6%)	301 (62.7%)	
Public	488 (37.9%)	66 (50.0%)	249 (36.8%)	173 (36.0%)	
Other/Unknown	38 (2.9%)	1 (0.8%)	31 (4.6%)	6 (1.3%)	
Median household income					0.029
Less than $40,000	302 (23.5%)	38 (28.8%)	167 (24.7%)	97 (20.2%)	
$40,000–$89,999	884 (68.6%)	86 (65.2%)	449 (66.4%)	349 (72.7%)	
$90,000 or more	80 (6.2%)	8 (6.1%)	42 (6.2%)	30 (6.3%)	
Unknown	22 (1.7%)	0 (0%)	18 (2.7%)	4 (0.8%)	
*Co-morbid Diagnoses*^*a*^					
*Medical Diagnoses*	1045 (81.1%)	107 (81.1%)	530 (78.4%)	408 (85.0%)	0.019
Malnutrition	969 (75.2%)	104 (78.8%)	475 (70.3%)	390 (81.3%)	< 0.001
Gastro-esophageal reflux disease	185 (14.4%)	13 (9.9%)	111 (16.4%)	61 (12.7%)	0.061
Food allergy	107 (8.3%)	6 (4.6%)	56 (8.3%)	45 (9.4%)	0.205
Eosinophilic esophagitis	36 (2.8%)	0 (0%)	25 (3.7%)	11 (2.3%)	0.043
Celiac disease	27 (2.1%)	1 (0.8%)	13 (1.9%)	13 (2.7%)	0.345
Inflammatory bowel disease	8 (0.6%)	1 (0.8%)	5 (0.7%)	2 (0.4%)	0.772
*Psychiatric Diagnoses*	1053 (81.8%)	107 (81.1%)	554 (82.0%)	392 (81.7%)	0.969
Any anxiety disorder	947 (73.5%)	94 (71.2%)	503 (74.4%)	350 (72.9%)	0.696
Generalized anxiety disorder	346 (26.9%)	29 (22.0%)	195 (28.9%)	122 (25.4%)	0.176
Obsessive–compulsive disorder	130 (10.1%)	10 (7.6%)	81 (12.0%)	39 (8.1%)	0.060
Phobic anxiety disorder	129 (10.0%)	6 (4.6%)	58 (8.6%)	65 (13.5%)	0.002
Adjustment disorder	121 (9.4%)	19 (14.4%)	70 (10.4%)	32 (6.7%)	0.012
Panic disorder	86 (6.7%)	8 (6.1%)	49 (7.3%)	29 (6.0%)	0.689
Other anxiety disorder	540 (41.9%)	53 (40.2%)	265 (39.2%)	222 (46.3%)	0.052
Depressive disorder	415 (32.2%)	49 (37.1%)	227 (33.6%)	139 (29.0%)	0.113
Attention-deficit hyperactivity disorder (ADHD)	173 (13.4%)	16 (12.1%)	72 (10.7%)	85 (17.7%)	0.002
Autism spectrum disorder	113 (8.8%)	9 (6.8%)	58 (8.6%)	46 (9.6%)	0.590

*SD* standard deviation

^a^Co-morbid diagnoses are not mutually exclusive; identified by ICD-10 diagnosis codes (see Additional File 1: Supplementary Appendix A)

Hospital utilization overall and by hospital volume can be found in Table 2. Median LOS was 7 days (IQR = 8; range 1–197). Cumulative total bed-days across all hospitals was 12,407 days. LOS differed significantly by hospital volume with shorter median stays at low volume hospitals and longer median stays at high volume hospitals (*p* < 0.001). Median costs per discharge were $16,583 (IQR = $18,115; range $1020–$448,858). Cumulative total costs across all hospitals were $28,997,294. Costs were lowest at low volume hospitals and highest at high volume hospitals (*p* < 0.001). GI imaging procedures and enteral tube feeding were common while surgical procedures were uncommon. By 30 days, 8.5% had been readmitted and readmissions did not differ by hospital volume (*p* = 0.883).

**Table 2:** Hospital utilization for patients hospitalized with ARFID at 22 PHIS hospitals (N = 1288)

	n (%)				*p* value
	Overall (N = 1288; 22 hospitals)	Hospital ARFID volume			
		Low (n = 132; 5 hospitals)	Medium (n = 676; 13 hospitals)	High (n = 480; 4 hospitals)	
Length of stay (LOS), *median (IQR)*	7.0 (8.0)	6.0 (6.5)	7.0 (7.0)	9.0 (7.0)	< 0.001
Length of stay category					< 0.001
< 4 days	247 (19.2%)	30 (22.7%)	152 (22.5%)	65 (13.5%)	
4–6 days	302 (23.5%)	38 (28.8%)	181 (26.8%)	83 (17.3%)	
7–14 days	504 (39.1%)	49 (37.1%)	225 (33.3%)	230 (47.9%)	
≥ 15 days	235 (18.3%)	15 (11.4%)	118 (17.5%)	102 (21.3%)	
ICU utilization	27 (2.1%)	3 (2.3%)	19 (2.8%)	5 (1.0%)	0.116
Enteral tube feeding^a^	386 (30.0%)	23 (17.4%)	246 (36.4%)	117 (24.4%)	< 0.001
GI imaging procedures^b^	556 (43.2%)	49 (37.1%)	308 (45.6%)	199 (41.5%)	0.128
Endoscopy	195 (15.1%)	19 (14.4%)	103 (15.2%)	73 (15.2%)	0.969
Colonoscopy	49 (3.8%)	4 (3.0%)	28 (4.1%)	17 (3.5%)	0.772
Other GI imaging^c^	498 (38.7%)	45 (34.1%)	283 (41.9%)	170 (35.4%)	0.045
Any surgical procedure	166 (12.9%)	17 (12.9%)	100 (14.8%)	49 (10.2%)	0.072
Costs ($), *median (IQR)*^d^	$16,583 ($18,115)	$14,247 ($17,264)	$14,955 ($16,638)	$18,971 ($18,517)	< 0.001
Readmissions within 30-days	109 (8.5%)	11 (8.3%)	55 (8.1%)	43 (9.0%)	0.883

*IQR* interquartile range; *ICU* intensive care unit; *GI* gastroenterological

^a^Identified by ICD-10 procedures codes and/or Clinical Transaction Classification (CTC) codes (see Additional File 1: Supplementary Appendix B)

^b^Not mutually exclusive; identified by ICD-10 procedure codes and/or Clinical Transaction Classification (CTC) codes (see Additional File 1: Supplementary Appendix C)

^c^Includes GI fluoroscopy, x-ray, ultrasound, MRI, CT scan, bone density, planar. See Additional File 1: Supplementary Appendix C for full listing

^d^Total standardized costs

Among the 109 patients readmitted within 30 days, there were a total of 112 readmissions. The median time from discharge to first readmission was 12.0 days (IQR = 14.0). Hospital utilization during the N = 112 readmissions is presented in Table 3. Median LOS was 6.0 days (IQR = 9.0) with a cumulative total of 993 bed-days. Similar to index admissions, enteral feeding and GI imaging procedures were common. Median costs were $12,475 (IQR = $20,321) with cumulative total costs of $2.3 million. The top reasons for readmission based on principal diagnosis code were ARFID (43%; n = 48), another eating disorder (11%; n = 12; n = 7 of which were anorexia nervosa) or malnutrition (6%; n = 7), signs and symptoms likely related to ARFID including dehydration or other lab abnormalities (9%; n = 10), other gastrointestinal diagnosis (7%; n = 8), enteral feeding-related (6%; n = 7), and other diagnoses likely unrelated to ARFID such as infections (8%; n = 9). Of note, there was n = 1 readmission for pneumothorax and n = 1 acute kidney failure, severe outcomes we considered possibly related to ARFID. Median LOS during readmissions differed significantly among the top 3 reasons for readmission (*p* = 0.003) with longer stays among those related to ARFID (median = 8.5; IQR = 13.0) or another eating disorder (median = 7.0; IQR = 11.0) and shorter stays for those with a principal diagnosis for signs and symptoms likely related to ARFID (median = 2.5; IQR = 4.0). 40 (36%) had a principal procedure during readmission and of these, 26 (65%) were enteral feeding tube placement, removal, or use, 7 (18%) GI imaging or biopsy, and 7 (18%) other procedures likely unrelated to ARFID. The full listing of principal diagnosis codes, procedure codes, and classifications during readmissions can be found in Additional File 2: Table S1.

**Table 3:** Hospital utilization during 30-day readmissions for patients with ARFID at 22 PHIS hospitals (N = 112 readmissions)

	n (%)
Length of stay, *median (IQR)*	6.0 (9.0)
ICU admission	4 (4%)
Enteral feeding^a^	46 (41%)
GI imaging procedures^b^	45 (40%)
Any surgical procedure	11 (10%)
Costs ($), *median (IQR)*^c^	$12,475 ($20,321)

*ICU* intensive care unit; *GI* gastroenterologic

^a^Identified by ICD-10 procedures codes and/or Clinical Transaction Classification (CTC) codes (see Additional File 1: Supplementary Appendix B)

^b^Identified by ICD-10 procedure codes and/or Clinical Transaction Classification (CTC) codes (see Additional File 1: Supplementary Appendix C)

^c^Total standardized costs

### Factors associated with hospital utilization (Aim 3)

**Fig. 2:**
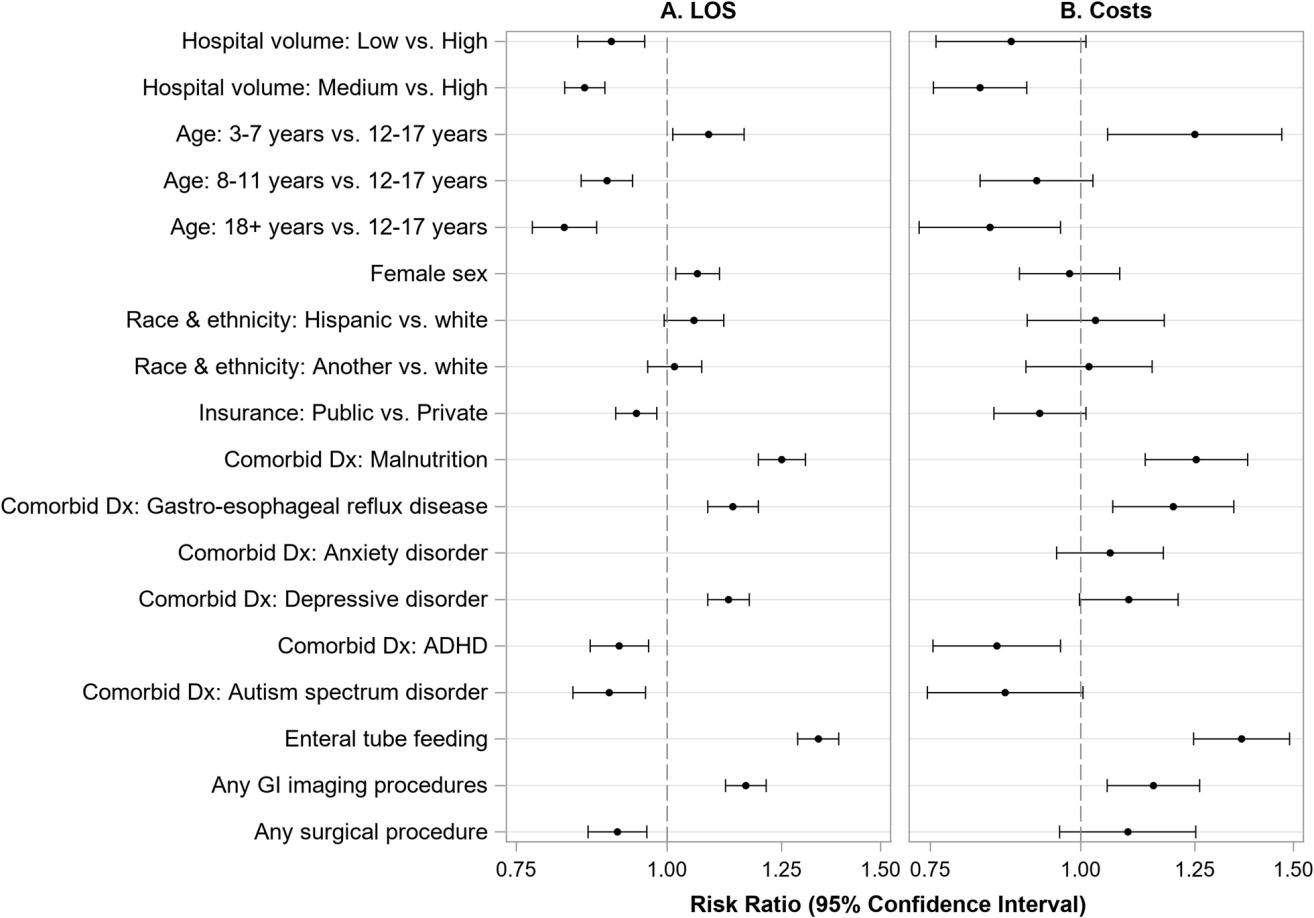
Factors associated with **A** LOS and **B** costs for patients hospitalized with ARFID. Among N = 1288 patients hospitalized with ARFID at 22 PHIS hospitals. Results from adjusted regression models using Poisson regression for LOS (**A**) and Gamma regression for costs (**B**). Abbreviations: LOS—length of stay; Dx—diagnosis; ADHD—attention-deficit hyperactivity disorder; GI—gastroenterologic

Factors associated with index admissions LOS and costs from adjusted models can be found in Fig. 2. Hospital volume, age, sex, race and ethnicity, insurance payor, co-morbid diagnoses, and procedures during admission were associated with LOS (Fig. 2A). Patients discharged from low and medium volume hospitals were likely to have shorter stays relative to high volume hospitals (low aRR = 0.90; 95% CI 0.84–0.96; medium aRR = 0.85; 95% CI 0.82–0.89). Compared to adolescent age patients (12–17 years), patients aged 8–11 years (aRR = 0.89; 95% CI 0.85–0.93) or over 18 years (aRR = 0.82; 95% CI 0.77–0.87) had shorter stays while those aged 3–7 years (aRR = 1.11; 95% CI 1.03–1.18) had longer stays. Females also had longer stays compared to males (aRR = 1.05; 95% CI 1.01–1.10). Relative to white non-Hispanic patients, Hispanic patients had longer stays (aRR = 1.06; 95% CI 1.00–1.13), though there was no significant difference for patients of another race (aRR = 1.03; 95% CI 0.98–1.08). Patients with public insurance had shorter stays compared to those with private insurance (aRR = 0.94; 95% CI 0.91–0.98). Co-morbid diagnosis of ADHD (aRR = 0.91; 95% CI 0.86–0.96) or autism spectrum disorder (aRR = 0.89; 95% CI 0.83–0.96) was associated with shorter stays while malnutrition (aRR = 1.24; 95% CI 1.19–1.30), gastro-esophageal reflux disease (aRR = 1.14; 95% CI 1.08–1.19), anxiety disorders (aRR = 1.08; 95% CI 1.03–1.13) or depressive disorders (aRR = 1.11; 95% CI 1.06–1.15) were associated with longer stays. Enteral tube feeding (aRR = 1.33; 95% CI 1.28–1.39) and GI imaging procedures (aRR = 1.16; 95% CI 1.12–1.21) during admission were also associated with longer stays while any surgical procedures during admission (aRR = 0.90; 95% CI 0.85–0.96) was associated with shorter stays.

An adjusted model examining costs indicated similar findings as LOS (Fig. 2B) with significantly lower costs among discharges from medium volume hospitals compared to high volume hospitals (aRR = 0.82; 95% CI 0.75–0.90), higher costs among the youngest patients aged 3–7 years (aRR = 1.25; 95% CI 1.05–1.47) and lower costs among patients age 18 or above (aRR = 0.84; 95% CI 0.73–0.96) compared to patients aged 12–17 years, higher costs for co-morbid diagnoses of malnutrition (aRR = 1.25; 95% CI 1.13–1.38) and gastro-esophageal reflux disease (aRR = 1.20; 95% CI 1.06–1.34), lower costs among patients with an ADHD diagnosis (aRR = 0.85; 95% CI 0.75–0.96), and higher costs associated with enteral tube feeding (aRR = 1.36; 95% CI 1.24–1.50) and GI imaging procedures (aRR = 1.15; 95% CI 1.05–1.26).

**Fig. 3:**
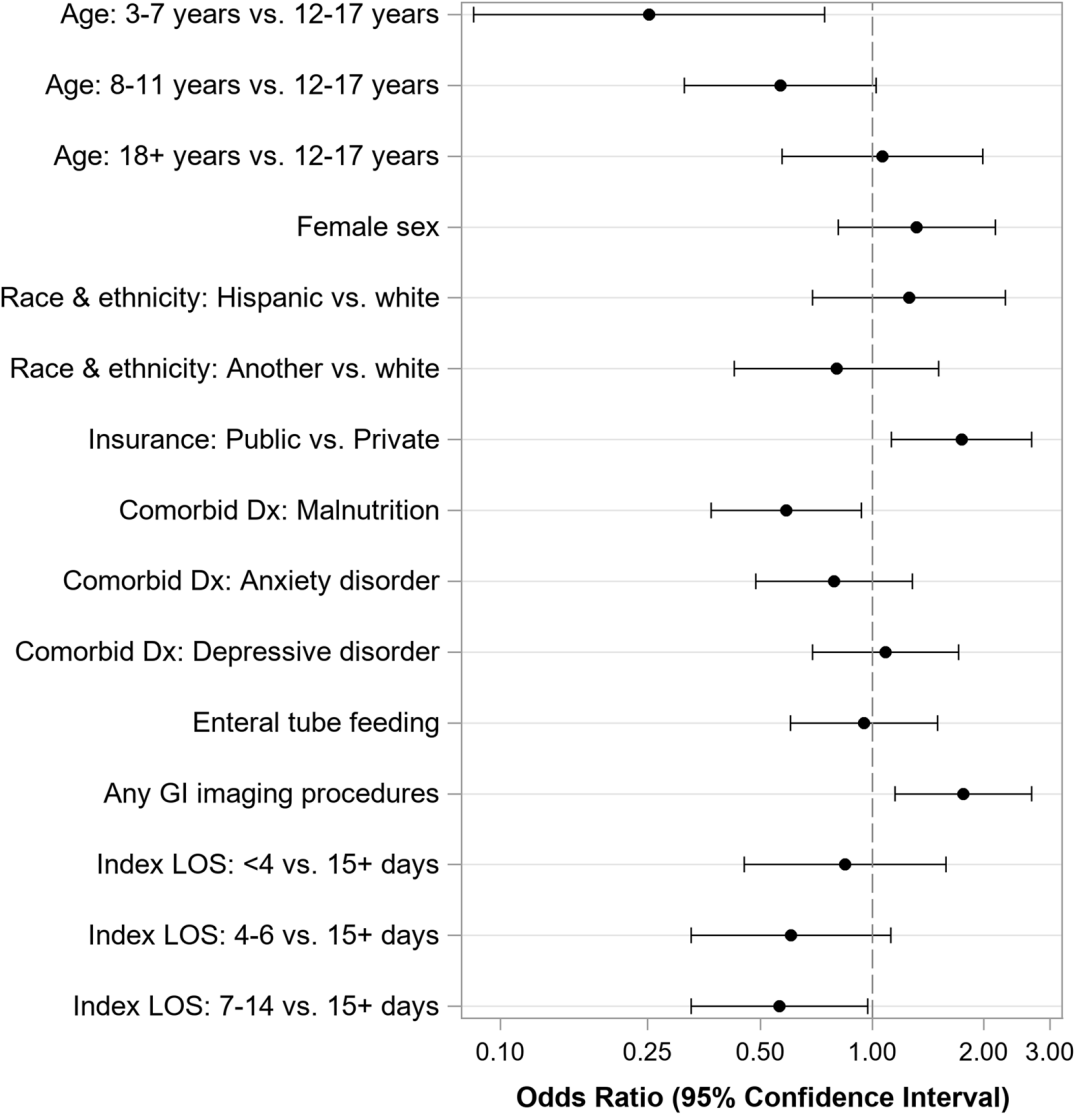
Factors associated with 30-day readmission for patients hospitalized with ARFID. Among N = 1288 patients hospitalized with ARFID at 22 PHIS hospitals. Results from adjusted logistic regression model. Abbreviations: LOS—length of stay; Dx—diagnosis; GI—gastroenterologic

Factors associated with 30-day readmission can be found in Fig. 3. Compared to adolescent age patients (12–17 years), patients aged 3–7 years had a lower odds of readmission (aOR = 0.25; 95% CI 0.08–0.75). A diagnosis of malnutrition during index admission was also associated with lower odds of readmission (aOR = 0.59; 95% CI 0.37–0.94), while public insurance (aOR = 1.74; 95% CI 1.13–2.68) and GI imaging procedures during index admission (aOR = 1.76; 95% CI 1.15–2.68) were associated with increased odds of 30-day readmissions. Index stays of 1–2 weeks were associated with a lower odds of readmission (aOR = 0.56; 95% CI 0.33–0.97) compared to stays longer than 2 weeks.

## Discussion

Since inclusion in the DSM-5 and ICD-10, the use of the ARFID diagnosis code has steadily increased across 44 pediatric tertiary care hospitals in the U.S. This may be secondary to an improved recognition of the disorder but also in part to the increased incidence and worsening severity of feeding and eating disorders following the onset of the COVID-19 pandemic and paralleling increases in other mental health disorders including anxiety and depression [13–17, 32]. As ARFID is a relatively newly described condition [1, 2, 4, 5], these findings suggest a growing need for both treatment options and provider awareness of this complex feeding and eating disorder. Our analysis also indicated that ARFID hospitalizations and readmissions are long and costly, particularly at high volume hospitals and in patients with medical and psychiatric co-morbidities.

Compared to prior single center studies of patients with anorexia nervosa hospitalized for medical stabilization, our findings indicate that patients with ARFID have comparable or shorter lengths of stay and similar costs, though large scale studies using PHIS or other administrative data sources allowing for direct comparisons across eating disorder diagnoses are lacking [33, 34]. Similar to previously described ARFID cohorts, we found that most patients had co-morbid mental health diagnoses, particularly anxiety, while relatively few had co-morbid GI disorders [1, 12]. Hospital stays for ARFID were long and costly, with over twelve thousand cumulative bed-days and costs of nearly $29 million during the entire study period. In 2021, the most recent full calendar year included in our study period, there were over 3400 cumulative bed-days with a cumulative hospital costs of $8.3 million.

Contrary to our hypothesis that hospitals treating more inpatients with ARFID may be more efficient, we found that hospitals with higher volumes of patients with ARFID also had higher utilization including longer stays and higher costs, even using costs that had been standardized to account for between-and-within hospital variation as well as regional variation [31]. This may be explained by higher patient complexity at higher volume hospitals, though it could also be due to variation in either practice patterns or hospital billing practices. Interestingly, we did not find that high volume hospitals had patients with a universally increased burden of co-morbid conditions, though certain conditions were most prevalent among high volume hospitals (e.g., malnutrition). Thus, the increased costs may not be easily explained by overall increased complexity. Similarly, although overall utilization was higher, patients at high volume hospitals were less likely to receive enteral tube feeding or GI imaging procedures. While we hypothesized that higher volume hospitals with more experience treating patients with ARFID would be likely to have more efficient treatment approaches, our findings suggest this may not be the case, though hospital variation in utilization and outcomes for this population should be examined in more detail.

Unsurprisingly, we found GI imaging procedures and additional workup during admission were associated with longer stays and higher costs. Our findings also suggest that enteral tube feeding is associated with higher utilization with no impact on 30-day readmissions; however, there may certainly be benefits such as weight stabilization and/or improvements in micronutrient deficiencies that we were unable to explore and might justify the longer LOS and higher costs. Future studies should examine factors associated with enteral tube feeding as well as the time-to-initiation to determine if initiating such nutritional support earlier in the hospital course can reduce overall utilization or prevent readmissions.

Consistent with our hypothesis, co-morbid anxiety or depressive disorder diagnosis was associated with longer stays. This may be related to time needed for anxiety/depressive symptom stabilization and/or greater complexity in the discharge planning process when accounting for mental health treatment needs post discharge. Patients with a diagnosis of ADHD, however, had shorter stays compared to those without which may be explained, in part, by age differences in both ADHD prevalence and LOS. In our cohort, ADHD diagnosis was most common in older children and adolescents who were also more likely to have shorter stays. Future studies are needed to explore this finding and the relationship between ADHD diagnosis and hospital utilization.

Interestingly, we found differences in hospital utilization by race and ethnicity with longer stays for patients who were Hispanic ethnicity compared to white patients. There were no significant differences by race and ethnicity for other sociodemographic factors like insurance or sex, or clinical characteristics that may be related to severity such as co-morbid diagnoses, enteral feeding, or use of GI imaging procedures that could explain these differences in LOS. It is unknown what factors are driving higher utilization among Hispanic patients and this is an important area for future study to explore this as well as other issues related to health equity.

We found 30-day readmissions of 8.5% in our cohort, higher than readmission rates reported in other studies of patients with eating disorders [35, 36]. In adjusted analyses, there were differences in the likelihood of readmission by age, insurance, and diagnosis for malnutrition. Patients with public insurance were more likely to be readmitted within 30-days which may be explained by slightly shorter index stays among patients with public insurance compared to those with private insurance, though this finding should be explored in more detail given the potential implications related to access and health equity. We found malnutrition diagnosis at index admission was associated with a lower odds of readmission which may be explained by differences in severity and utilization during index admission. LOS was significantly longer for malnourished patients compared to those without a diagnosis for malnutrition which may indicate that more severely malnourished patients received more intensive treatment during index admission, reducing the risk of readmission. Future studies should explore interventions and treatments to reduce readmissions for this patient population.

During readmissions, average stays were just under one week and frequently related to enteral feeding. The majority of readmissions were for ARFID, another eating disorder or malnutrition, or symptoms we considered likely related to ARFID which likely reflects the complexity of caring for this patient population, but could indicate inadequate care received during index visit. We identified two readmissions for severe outcomes that may have been precipitated by ARFID (pneumothorax and kidney failure), highlighting the severity of the disease and its potential for catastrophic consequences [37]. Prior studies of patients with anorexia nervosa have established links between eating disorders and these conditions, and severely malnourished patients with ARFID may be similarly at risk for organ failure and dysfunction [38, 39]. However, we were unable to explore this further given the limitations of administrative data and we can only speculate that these readmissions are related to underlying ARFID rather than another etiology. We focused our analysis on short term readmissions within 30 days which may be more likely to be unplanned and attributable to the quality of care during initial hospitalization [40, 41]. Additionally, we describe all-cause readmissions and did not stratify by preventability [40, 41]. Future studies are needed to explore preventability of readmissions as well as those occurring later in the post-discharge period to better understand the mechanisms for readmissions in this patient population.

Our study has several limitations. First, PHIS hospitals may not be generalizable to all tertiary care pediatric hospitals or community hospitals. Second, administrative billing data may be incomplete with regard to co-morbidities and clinical factors. We identified patients with ARFID based on principal discharge diagnosis code which may underestimate the total ARFID population at PHIS hospitals. As a relatively new condition, it is likely ARFID is under-recognized and/or not billed for, leading to underestimates. Given the lack of a specific diagnostic code for ARFID prior to inclusion in ICD-10 in October 2017, we were unable to examine ARFID volume prior to this date in the context of administrative data across multiple hospitals as this would require extensive chart review to verify the diagnosis and exclude encounters related to other feeding disorders or behaviors inconsistent with ARFID. Third, readmissions in PHIS are underestimated as only readmissions to the same PHIS hospital are captured. Finally, there was no additional clinical information available including ARFID subtype or severity at presentation, as well as presenting weight, or change in weight or vital signs during admission. In addition, there was no other diagnostic information available including the criteria used to diagnose ARFID and/or the timing (prior to vs. during admission) or setting (i.e., outpatient, inpatient, another hospital or care setting) of initial diagnosis to determine how or when a patient was first diagnosed. Despite these limitations, PHIS data includes robust estimates of cost and is both timely and geographically diverse and our cohort included pediatric tertiary care hospitals across the United States.

## Conclusions

In a diverse cohort of pediatric hospitals, we found that hospitalizations for ARFID have increased over time since the inception of the diagnosis code indicating an increasing need for treatment options. Our results indicate hospital stays for ARIFD are long and costly with significant variation by hospital ARFID volume which may present opportunities for improvement and standardization of care. More than just ‘picky eating,’ ARFID is a serious feeding and eating disorder that can result in significant morbidity [1, 42]. ARFID is distinct from other restrictive eating disorders as it is not related to issues with body image or a desire for thinness making it difficult to identify and manage [1]. Given the increasing volume of ARFID hospitalizations, along with high costs and long LOS for these hospitalizations, it is imperative that effective treatment strategies are identified, including approaches to medical workup, timing and use of enteral feeding tubes, and interventions to reduce readmissions.

### Supplementary Information

**Additional file 1.** Codes to identify co-morbid diagnoses, enteral tube feeding, and GI imaging procedures.

**Additional file 2.** Reasons for readmission using principal diagnosis and procedure codes.

## Data Availability

The datasets analyzed in this study are not publicly available as they were acquired through the Pediatric Health Information System which prohibits data sharing outside of its member hospitals.

## Abbreviations

ARFID: Avoidant/restrictive food intake disorder
DSM-5: *Diagnostic and Statistical Manual of Mental Disorders*
ICD-10: *International Classification of Diseases, 10th Revision*
PHIS: Pediatric Health Information System
CTC: Clinical Transaction Classification
LOS: Length of stay
ADHD: Attention-deficit hyperactivity disorders
GI: Gastroenterologic or gastrointestinal
SD: Standard deviation
IQR: Interquartile range
aRR: Adjusted risk ratio
aOR: Adjusted odds ratio
CI: Confidence interval
