# Supplementary material for: Pediatric hospital utilization for patients with avoidant restrictive food intake disorder
Source: J Eat Disord. 2024 Mar 25;12:42. doi: 10.1186/s40337-024-00996-z (PMC10962111; doi:10.1186/s40337-024-00996-z)
Supplement: Supplementary file 1 — Additional file 1. Codes to identify co-morbid diagnoses, enteral tube feeding, and GI imaging procedures. [file 40337_2024_996_MOESM1_ESM.docx]

**Supplementary Appendix A.** ICD-10 diagnosis codes used to define co-morbid diagnoses.

| **Code** | **Description** | **Grouping** |
| --- | --- | --- |
| *Medical Diagnoses* | | |
| K20.0 | Eosinophilic esophagitis | Eosinophilic esophagitis |
| K21.9 | Gastro-esophageal reflux disease without esophagitis | Gastro-esophageal reflux disease (GERD) |
| K21.0 | Gastro-esophageal reflux disease with esophagitis |  |
| K50 | Crohn's disease [regional enteritis] | Inflammatory bowel disease |
| K51 | Ulcerative colitis |  |
| K90.0 | Celiac disease | Celiac disease |
| Z91.01 | Food allergy status | Food allergy |
| Z68.51 | Body mass index [BMI] pediatric, less than 5th percentile for age | Malnutrition |
| Z68.1 | Body mass index [BMI] 19.9 or less, adult |  |
| E41 | Nutritional marasmus |  |
| E43 | Unspecified severe protein-calorie malnutrition |  |
| E44.0 | Moderate protein-calorie malnutrition |  |
| E44.1 | Mild protein-calorie malnutrition |  |
| E46 | Unspecified protein-calorie malnutrition |  |
| *Psychiatric Diagnoses* | | |
| F90 | Attention-deficit hyperactivity disorders | Attention-deficit hyperactivity disorders (ADHD) |
| F84.0 | Autistic disorder | Autism spectrum disorder |
| F41.1 | Generalized anxiety disorder | Generalized anxiety disorder |
| F40 | Phobic anxiety disorders | Phobic anxiety disorders |
| F41.0 | Panic disorder | Panic disorder |
| F42 | Obsessive-compulsive disorder | Obsessive-compulsive disorder (OCD) |
| F43 | Reaction to severe stress, and adjustment disorders | Adjustment disorders |
| F41.3 | Other mixed anxiety disorders | Other anxiety disorders |
| F41.8 | Other specified anxiety disorders |  |
| F41.9 | Anxiety disorder, unspecified |  |
| F44 | Dissociative and conversion disorders |  |
| F45 | Somatoform disorders |  |
| F48 | Other nonpsychotic mental disorders |  |
| F93.0 | Separation anxiety disorder of childhood |  |
| F32 | Depressive episode | Depressive disorder |
| F33 | Major depressive disorder, recurrent |  |
| F34.1 | Dysthymic disorder |  |

**Supplementary Appendix B.** ICD-10 procedure codes and Clinical Transaction Classification (CTC) codes used to define enteral tube feeding.

| **Type** | **Code** | **Description** |
| --- | --- | --- |
| ICD-10 Procedure | 3E0G76Z | Introduction of Nutritional into Up GI, Via Opening |
| ICD-10 Procedure | 3E0H76Z | Introduction of Nutritional into Low GI, Via Opening |
| ICD-10 Procedure | 3E0G36Z | Introduction of Nutritional into Up GI, Perc Approach |
| ICD-10 Procedure | 0DH64UZ | Insertion of Feeding Device into Stomach, Percutaneous Endoscopic Approach |
| ICD-10 Procedure | 0DH67UZ | Insertion of Feeding Device into Stomach, Via Natural or Artificial Opening |
| ICD-10 Procedure | 0DH63UZ | Insertion of Feeding Device into Stomach, Percutaneous Approach |
| ICD-10 Procedure | 0DHA7UZ | Insertion of Feeding Device into Jejunum, Via Natural or Artificial Opening |
| ICD-10 Procedure | 0DH68UZ | Insertion of Feeding Device into Stomach, Via Natural or Artificial Opening Endoscopic |
| ICD-10 Procedure | 0D20XUZ | Change Feeding Device in Upper Intestinal Tract, External Approach |
| ICD-10 Procedure | 0DH673Z | Insertion of Infusion Device into Stomach, Via Natural or Artificial Opening |
| ICD-10 Procedure | 0D2DXUZ | Change Feeding Device in Lower Intestinal Tract, External Approach |
| ICD-10 Procedure | 0DHA3UZ | Insertion of Feeding Device into Jejunum, Percutaneous Approach |
| ICD-10 Procedure | 0DH84UZ | Insertion of Feeding Device into Small Intestine, Percutaneous Endoscopic Approach |
| ICD-10 Procedure | 0DHA8UZ | Insertion of Feeding Device into Jejunum, Via Natural or Artificial Opening Endoscopic |
| ICD-10 Procedure | 0DPD7UZ | Removal of Feeding Device from Lower Intestinal Tract, Via Natural or Artificial Opening |
| ICD-10 Procedure | 0DPD8UZ | Removal of Feeding Device from Lower Intestinal Tract, Via Natural or Artificial Opening Endoscopic |
| ICD-10 Procedure | 0DH97UZ | Insertion of Feeding Device into Duodenum, Via Natural or Artificial Opening |
| CTC, Clinical | 541131 | Nasogastric tube placement |
| CTC, Clinical | 541141 | Enterostomal service |
| CTC, Supply | 255221 | Feeding tube and accessory |
| CTC, Supply | 244711 | Gastrostomy feeding tube |
| CTC, Supply | 244713 | Gastrojejunostomy feeding tube |
| CTC, Other services | 620100 | Adult enteral products |
| CTC, Other services | 620105 | Pediatric enteral products |

**Supplementary Appendix C.** ICD-10 procedure codes and Clinical Transaction Classification (CTC) codes used to define GI imaging procedures.

| **Type** | **Code** | **Description** | **Grouping** |
| --- | --- | --- | --- |
| ICD-10 Procedure | 0DBN8ZX | Excision of Sigmoid Colon, Via Natural or Artificial Opening Endoscopic, Diagnostic | **Colonoscopy** |
| ICD-10 Procedure | 0DBM8ZX | Excision of Descending Colon, Via Natural or Artificial Opening Endoscopic, Diagnostic |  |
| ICD-10 Procedure | 0DBP8ZX | Excision of Rectum, Via Natural or Artificial Opening Endoscopic, Diagnostic |  |
| ICD-10 Procedure | 0DBL8ZX | Excision of Transverse Colon, Via Natural or Artificial Opening Endoscopic, Diagnostic |  |
| ICD-10 Procedure | 0DBK8ZX | Excision of Ascending Colon, Via Natural or Artificial Opening Endoscopic, Diagnostic |  |
| ICD-10 Procedure | 0DBF8ZX | Excision of Right Large Intestine, Via Natural or Artificial Opening Endoscopic, Diagnostic |  |
| ICD-10 Procedure | 0DBE8ZX | Excision of Large Intestine, Via Natural or Artificial Opening Endoscopic, Diagnostic |  |
| ICD-10 Procedure | 0DBC8ZX | Excision of Ileocecal Valve, Via Natural or Artificial Opening Endoscopic, Diagnostic |  |
| ICD-10 Procedure | 0DBG8ZX | Excision of Left Large Intestine, Via Natural or Artificial Opening Endoscopic, Diagnostic |  |
| ICD-10 Procedure | 0DCP7ZZ | Extirpation of Matter from Rectum, Via Natural or Artificial Opening |  |
| ICD-10 Procedure | 0DJD8ZZ | Inspection of Lower Intestinal Tract, Via Natural or Artificial Opening Endoscopic |  |
| ICD-10 Procedure | 0D9B8ZX | Drainage of Ileum, Via Natural or Artificial Opening Endoscopic, Diagnostic |  |
| ICD-10 Procedure | 0D9H8ZX | Drainage of Cecum, Via Natural or Artificial Opening Endoscopic, Diagnostic |  |
| ICD-10 Procedure | 0D9L8ZX | Drainage of Transverse Colon, Via Natural or Artificial Opening Endoscopic, Diagnostic |  |
| ICD-10 Procedure | 0D9M8ZX | Drainage of Descending Colon, Via Natural or Artificial Opening Endoscopic, Diagnostic |  |
| ICD-10 Procedure | 0D9N8ZX | Drainage of Sigmoid Colon, Via Natural or Artificial Opening Endoscopic, Diagnostic |  |
| ICD-10 Procedure | 0D9P8ZX | Drainage of Rectum, Via Natural or Artificial Opening Endoscopic, Diagnostic |  |
| ICD-10 Procedure | 0DBQ8ZX | Excision of Anus, Via Natural or Artificial Opening Endoscopic, Diagnostic |  |
| ICD-10 Procedure | 0DDB8ZX | Extraction of Ileum, Via Natural or Artificial Opening Endoscopic, Diagnostic |  |
| ICD-10 Procedure | 0DDC8ZX | Extraction of Ileocecal Valve, Via Natural or Artificial Opening Endoscopic, Diagnostic |  |
| ICD-10 Procedure | 0DDH8ZX | Extraction of Cecum, Via Natural or Artificial Opening Endoscopic, Diagnostic |  |
| ICD-10 Procedure | 0DDK8ZX | Extraction of Ascending Colon, Via Natural or Artificial Opening Endoscopic, Diagnostic |  |
| ICD-10 Procedure | 0DDL8ZX | Extraction of Transverse Colon, Via Natural or Artificial Opening Endoscopic, Diagnostic |  |
| ICD-10 Procedure | 0DDM8ZX | Extraction of Descending Colon, Via Natural or Artificial Opening Endoscopic, Diagnostic |  |
| ICD-10 Procedure | 0DDN8ZX | Extraction of Sigmoid Colon, Via Natural or Artificial Opening Endoscopic, Diagnostic |  |
| ICD-10 Procedure | 0DDP8ZX | Extraction of Rectum, Via Natural or Artificial Opening Endoscopic, Diagnostic |  |
| ICD-10 Procedure | 0DJD3ZZ | Inspection of Lower Intestinal Tract, Percutaneous Approach |  |
| ICD-10 Procedure | 0DBB8ZX | Excision of Ileum, Via Natural or Artificial Opening Endoscopic, Diagnostic |  |
| ICD-10 Procedure | 0DBH8ZX | Excision of Cecum, Via Natural or Artificial Opening Endoscopic, Diagnostic |  |
| ICD-10 Procedure | 0DB98ZX | Excision of Duodenum, Via Natural or Artificial Opening Endoscopic, Diagnostic | **Endoscopy**  **Endoscopy, cont.** |
| ICD-10 Procedure | 0DB68ZX | Excision of Stomach, Via Natural or Artificial Opening Endoscopic, Diagnostic |  |
| ICD-10 Procedure | 0DB38ZX | Excision of Lower Esophagus, Via Natural or Artificial Opening Endoscopic, Diagnostic |  |
| ICD-10 Procedure | 0DB78ZX | Excision of Stomach, Pylorus, Via Natural or Artificial Opening Endoscopic, Diagnostic |  |
| ICD-10 Procedure | 0DB18ZX | Excision of Upper Esophagus, Via Natural or Artificial Opening Endoscopic, Diagnostic |  |
| ICD-10 Procedure | 0DB58ZX | Excision of Esophagus, Via Natural or Artificial Opening Endoscopic, Diagnostic |  |
| ICD-10 Procedure | 0DB28ZX | Excision of Middle Esophagus, Via Natural or Artificial Opening Endoscopic, Diagnostic |  |
| ICD-10 Procedure | 0DD98ZX | Extraction of Duodenum, Via Natural or Artificial Opening Endoscopic, Diagnostic |  |
| ICD-10 Procedure | 0DD68ZX | Extraction of Stomach, Via Natural or Artificial Opening Endoscopic, Diagnostic |  |
| ICD-10 Procedure | 0DD38ZX | Extraction of Lower Esophagus, Via Natural or Artificial Opening Endoscopic, Diagnostic |  |
| ICD-10 Procedure | 0DJ08ZZ | Inspection of Upper Intestinal Tract, Via Natural or Artificial Opening Endoscopic |  |
| ICD-10 Procedure | 0CJS8ZZ | Inspection of Larynx, Via Natural or Artificial Opening Endoscopic |  |
| ICD-10 Procedure | 0D9670Z | Drainage of Stomach with Drainage Device, Via Natural or Artificial Opening |  |
| ICD-10 Procedure | 0DD18ZX | Extraction of Upper Esophagus, Via Natural or Artificial Opening Endoscopic, Diagnostic |  |
| ICD-10 Procedure | 0DD58ZX | Extraction of Esophagus, Via Natural or Artificial Opening Endoscopic, Diagnostic |  |
| ICD-10 Procedure | 3E0G8GC | Introduction of Other Therapeutic Substance into Up GI, Endo |  |
| ICD-10 Procedure | 0CJY8ZZ | Inspection of Mouth and Throat, Via Natural or Artificial Opening Endoscopic |  |
| ICD-10 Procedure | 0DJ07ZZ | Inspection of Upper Intestinal Tract, Via Natural or Artificial Opening |  |
| ICD-10 Procedure | 4A0B7BZ | Measurement of Gastrointestinal Pressure, Via Natural or Artificial Opening |  |
| ICD-10 Procedure | 4A1B88Z | Monitoring of Gastrointestinal Motility, Via Natural or Artificial Opening Endoscopic |  |
| ICD-10 Procedure | 0BC18ZZ | Extirpation of Matter from Trachea, Via Natural or Artificial Opening Endoscopic |  |
| ICD-10 Procedure | 0D778ZZ | Dilation of Stomach, Pylorus, Via Natural or Artificial Opening Endoscopic |  |
| ICD-10 Procedure | 0D958ZX | Drainage of Esophagus, Via Natural or Artificial Opening Endoscopic, Diagnostic |  |
| ICD-10 Procedure | 0D9680Z | Drainage of Stomach with Drainage Device, Via Natural or Artificial Opening Endoscopic |  |
| ICD-10 Procedure | 0D968ZX | Drainage of Stomach, Via Natural or Artificial Opening Endoscopic, Diagnostic |  |
| ICD-10 Procedure | 0D998ZX | Drainage of Duodenum, Via Natural or Artificial Opening Endoscopic, Diagnostic |  |
| ICD-10 Procedure | 0DB38ZZ | Excision of Lower Esophagus, Via Natural or Artificial Opening Endoscopic |  |
| ICD-10 Procedure | 0DB48ZX | Excision of Esophagogastric Junction, Via Natural or Artificial Opening Endoscopic, Diagnostic |  |
| ICD-10 Procedure | 0DB88ZX | Excision of Small Intestine, Via Natural or Artificial Opening Endoscopic, Diagnostic |  |
| ICD-10 Procedure | 0DD28ZX | Extraction of Middle Esophagus, Via Natural or Artificial Opening Endoscopic, Diagnostic |  |
| ICD-10 Procedure | 0DD78ZX | Extraction of Stomach, Pylorus, Via Natural or Artificial Opening Endoscopic, Diagnostic |  |
| ICD-10 Procedure | 0DH982Z | Insertion of Monitoring Device into Duodenum, Via Natural or Artificial Opening Endoscopic |  |
| ICD-10 Procedure | 0DJ68ZZ | Inspection of Stomach, Via Natural or Artificial Opening Endoscopic |  |
| ICD-10 Procedure | 0F9G4ZZ | Drainage of Pancreas, Percutaneous Endoscopic Approach |  |
| ICD-10 Procedure | 4A0B78Z | Measurement of Gastrointestinal Motility, Via Natural or Artificial Opening |  |
| ICD-10 Procedure | BD15YZZ | Fluoroscopy of Upper GI using Other Contrast | **Other GI or nutrition Imaging**  **Other GI or nutrition Imaging, cont.** |
| ICD-10 Procedure | BD15ZZZ | Fluoroscopy of Upper GI |  |
| ICD-10 Procedure | BD47ZZZ | Ultrasonography of Gastrointestinal Tract |  |
| ICD-10 Procedure | CF141ZZ | Planar Nuclear Medicine Imaging of Gallbladder using Technetium 99m (Tc-99m) |  |
| CTC, Imaging | 441311 | Abdomen supine x-ray |  |
| CTC, Imaging | 443122 | Stomach and upper GI fluoroscopy |  |
| CTC, Imaging | 441611 | Abdomen supine upright decubitus and cross-table x-ray |  |
| CTC, Imaging | 441141 | Upper abdomen ultrasound real-time |  |
| CTC, Imaging | 442222 | Esophagus fluoroscopy |  |
| CTC, Imaging | 443563 | Gastric emptying study Planar imaging |  |
| CTC, Imaging | 441122 | Upper abdomen fluoroscopy |  |
| CTC, Imaging | 427365 | Bone density dual photon absorption |  |
| CTC, Imaging | 441152 | Upper abdomen MRI |  |
| CTC, Imaging | 441051 | Abdomen and pelvis CT scan |  |
| CTC, Imaging | 441252 | Lower abdomen MRI |  |
| CTC, Imaging | 443222 | Upper GI with small bowel follow-through fluoroscopy |  |
| CTC, Imaging | 427111 | Bone age x-ray |  |
| CTC, Imaging | 464440 | Noninvasive vascular ultrasound of abdomen |  |
| CTC, Imaging | 457041 | Retroperitoneum ultrasound real-time |  |
| CTC, Imaging | 464445 | Noninvasive vascular Doppler of abdomen |  |
| CTC, Imaging | 445163 | Liver and biliary tree Planar imaging |  |
| CTC, Imaging | 440052 | Gastrointestinal MRI |  |
| CTC, Imaging | 441142 | Upper abdomen ultrasound B-mode |  |
| CTC, Imaging | 475051 | Abdomen arteriography CT scan |  |
| CTC, Imaging | 475052 | Abdomen arteriography MRI |  |
| CTC, Imaging | 442263 | Gastroesophageal reflux study Planar imaging |  |
| CTC, Imaging | 444022 | Small bowel fluoroscopy |  |
| CTC, Imaging | 444741 | Appendix ultrasound real-time |  |
| CTC, Imaging | 445141 | Liver and biliary tree ultrasound real-time |  |
| CTC, Imaging | 441151 | Upper abdomen CT scan |  |
| CTC, Imaging | 442363 | Esophageal transit study Planar imaging |  |
| CTC, Imaging | 444322 | Colon fluoroscopy |  |
| CTC, Imaging | 445241 | Gall bladder ultrasound real-time |  |
| CTC, Imaging | 445552 | Imaging guidance for ERCP MRI |  |
| CTC, Imaging | 457040 | Retroperitoneum ultrasound |  |
| CTC, Imaging | 496922 | Imaging guidance for removal of stone and foreign body of other site fluoroscopy |  |
