# Supplementary material for: Pediatric hospital utilization for patients with avoidant restrictive food intake disorder
Source: J Eat Disord. 2024 Mar 25;12:42. doi: 10.1186/s40337-024-00996-z (PMC10962111; doi:10.1186/s40337-024-00996-z)
Supplement: Supplementary file 2 — Additional file 2. Reasons for readmission using principal diagnosis and procedure codes. [file 40337_2024_996_MOESM2_ESM.docx]

**Supplementary Table 1.** Principal diagnosis and procedure codes (ICD-10) and classification during readmissions (N=112 readmissions).

| **Code** | **Description** | **n (%)** | **Grouping** |  |
| --- | --- | --- | --- | --- |
| *Principal diagnosis* | | | | |
| F50.82 | Avoidant/restrictive food intake disorder | 44 (39%) | ARFID |  |
| R62.51 | Failure to thrive (child) | 2 (2%) |  |  |
| R63.3 | Feeding difficulties | 2 (2%) |  |  |
| F50.01 | Anorexia nervosa, restricting type | 4 (4%) | Other eating disorder |  |
| F50.00 | Anorexia nervosa, unspecified | 3 (3%) |  |  |
| F50.9 | Eating disorder, unspecified | 3 (3%) |  |  |
| F50.89 | Other specified eating disorder | 2 (2%) |  |  |
| E86.0 | Dehydration | 3 (3%) | Signs and symptoms  likely related to ARFID |  |
| E16.2 | Hypoglycemia, unspecified | 1 (1%) |  |  |
| E87.6 | Hypokalemia | 1 (1%) |  |  |
| R00.0 | Tachycardia, unspecified | 1 (1%) |  |  |
| R11.10 | Vomiting, unspecified | 1 (1%) |  |  |
| R11.2 | Nausea with vomiting, unspecified | 1 (1%) |  |  |
| R55 | Syncope and collapse | 1 (1%) | Other diagnosis |  |
| R94.5 | Abnormal results of liver function studies | 1 (1%) |  |  |
| A41.9 | Sepsis, unspecified organism | 1 (1%) |  |  |
| D57.219 | Sickle-cell/hb-c disease with crisis, unspecified | 1 (1%) |  |  |
| E10.65 | Type 1 diabetes mellitus with hyperglycemia | 1 (1%) |  |  |
| G37.3 | Acute transverse myelitis in demyelinating disease of central nervous system | 1 (1%) |  |  |
| G40.409 | Other generalized epilepsy and epileptic syndromes, not intractable, without status epilepticus | 1 (1%) |  |  |
| G89.29 | Other chronic pain | 1 (1%) |  |  |
| G91.1 | Obstructive hydrocephalus | 1 (1%) |  |  |
| L03.316 | Cellulitis of umbilicus | 1 (1%) |  |  |
| N94.6 | Dysmenorrhea, unspecified | 1 (1%) |  |  |
| G43.A1 | Cyclical vomiting, in migraine, intractable | 1 (1%) | Other gastrointestinal |  |
| K20.0 | Eosinophilic esophagitis | 1 (1%) |  |  |
| K30 | Functional dyspepsia | 1 (1%) |  |  |
| K31.84 | Gastroparesis | 1 (1%) |  |  |
| K58.9 | Irritable bowel syndrome without diarrhea | 1 (1%) |  |  |
| K59.00 | Constipation, unspecified | 1 (1%) |  |  |
| K59.09 | Other constipation | 1 (1%) |  |  |
| K59.89 | Other specified functional intestinal disorders | 1 (1%) |  |  |
| Z46.89 | Encounter for fitting and adjustment of other specified devices | 2 (2%) | Enteral feeding-related |  |
| T85.628A | Displacement of other specified internal prosthetic devices, implants and grafts, initial encounter | 1 (1%) |  |  |
| T85.9XXA | Unspecified complication of internal prosthetic device, implant and graft, initial encounter | 1 (1%) |  |  |
| Z43.1 | Encounter for attention to gastrostomy | 1 (1%) |  |  |
| Z45.89 | Encounter for adjustment and management of other implanted devices | 1 (1%) |  |  |
| Z46.59 | Encounter for fitting and adjustment of other gastrointestinal appliance and device | 1 (1%) |  |  |
| E46 | Unspecified protein-calorie malnutrition | 3 (3%) | Malnutrition |  |
| E43 | Unspecified severe protein-calorie malnutrition | 2 (2%) |  |  |
| E44.0 | Moderate protein-calorie malnutrition | 2 (2%) |  |  |
|  |  |  |  |  |
|  |  |  |  |  |
|  |  |  |  |  |
| *Principal Diagnosis, cont.* | | | | |
| F44.9 | Dissociative and conversion disorder, unspecified | 2 (2%) | Other psychiatric or  mental health condition |  |
| F28 | Other psychotic disorder not due to a substance or known physiological condition | 1 (1%) |  |  |
| F33.2 | Major depressive disorder, recurrent severe without psychotic features | 1 (1%) |  |  |
| F44.5 | Conversion disorder with seizures or convulsions | 1 (1%) |  |  |
| F45.9 | Somatoform disorder, unspecified | 1 (1%) |  |  |
| R10.11 | Right upper quadrant pain | 1 (1%) | Abdominal pain |  |
| R10.84 | Generalized abdominal pain | 1 (1%) |  |  |
| R10.9 | Unspecified abdominal pain | 1 (1%) |  |  |
| J93.9 | Pneumothorax, unspecified | 1 (1%) | Outcome possibly  related to ARFID |  |
| N17.9 | Acute kidney failure, unspecified | 1 (1%) |  |  |
| *Principal Procedure* | | | | |
| 0DH67UZ | Insertion Of Feeding Device Into Stomach, Via Opening | 9 (23%) | Enteral feeding-related |  |
| 3E0G76Z | Introduction Of Nutritional Into Up Gi, Via Opening | 6 (15%) |  |  |
| 0DHA7UZ | Insertion Of Feeding Device Into Jejunum, Via Opening | 4 (10%) |  |  |
| 0DH64UZ | Insertion Of Feeding Device Into Stomach, Perc Endo Approach | 3 (8%) |  |  |
| 0DH63UZ | Insertion Of Feeding Device Into Stomach, Perc Approach | 2 (5%) |  |  |
| 0DH84UZ | Insert Of Feeding Dev Into Small Intest, Perc Endo Approach | 1 (3%) |  |  |
| 0DP67UZ | Removal Of Feeding Device From Stomach, Via Opening | 1 (3%) |  |  |
| 0DB98ZX | Excision Of Duodenum, Endo, Diagn | 4 (10%) | GI Imaging or biopsy |  |
| 0CJY8ZZ | Inspection Of Mouth And Throat, Endo | 1 (3%) |  |  |
| 0D9670Z | Drainage Of Stomach With Drainage Device, Via Opening | 1 (3%) |  |  |
| BD16YZZ | Fluoroscopy Of Upper Gi And Small Bowel Using Other Contrast | 1 (3%) |  |  |
| 00164ZB | Bypass Cereb Vent To Cereb Cistern, Perc Endo Approach | 1 (3%) | Other procedure |  |
| 009U3ZZ | Drainage Of Spinal Canal, Percutaneous Approach | 1 (3%) |  |  |
| 03HY32Z | Insertion Of Monitoring Device Into Up Art, Perc Approach | 1 (3%) |  |  |
| 0W9930Z | Drainage Of R Pleural Cav With Drain Dev, Perc Approach | 1 (3%) |  |  |
| 3E0T3BZ | Introduce Anesthetic In Periph Nrv, Plexi, Perc | 1 (3%) |  |  |
| 3E1B78Z | Irrigation Of Ear Using Irrigating Substance, Via Opening | 1 (3%) |  |  |
| F08Z4ZZ | Home Management Treatment | 1 (3%) |  |  |
